# Clinical Features of Children with COVID-19 Infection: Single-Center Experience

**DOI:** 10.5152/eurasianjmed.2022.21083

**Published:** 2022-06-01

**Authors:** Muhammet Akif Guler, Fuat Laloglu, Zerrin Orbak, Naci Ceviz, Ali Islek

**Affiliations:** 1 Division of Pediatric Nephrology, Department of Pediatrics, Atatürk University Faculty of Medicine, Erzurum, Turkey; 2 Division of Pediatric Cardiology, Department of Pediatrics, Atatürk University Faculty of Medicine, Erzurum, Turkey; 3 Division of Pediatric Endocrinology, Department of Pediatrics, Atatürk University Faculty of Medicine, Erzurum, Turkey; 4 Division of Pediatric Gastroenterology, Department of Pediatrics, Çukurova University Faculty of Medicine, Adana, Turkey

**Keywords:** Coronavirus, COVID-19, SARS-CoV-2

## Abstract

**Objective:** Compared to adult studies, there are few epidemiological and clinical reports on coronavirus disease 2019 in children. We aimed to present the demographic, epidemiological, and clinical findings of hospitalized pediatric coronavirus disease 2019 patients.

**Materials and Methods:** Patients aged 0–18 years who were hospitalized between March and July 2020 due to severe acute respiratory syndrome coronavirus 2 infection were evaluated retrospectively.

**Results:** The mean age was 90.2 ± 67.5 (7–24) months and 23 (51%) were female. Clinical presentation was asymptomatic in 15 cases (33.3%), mild/moderate in 26 cases (57.8%), and severe/critical in 4 cases (8.9%). Three (6.6%) of the patients had chronic medical conditions that placed them in the high-risk group for coronavirus disease 2019. The source of infection was household transmission in 29 cases (64.4%). The most common symptoms were cough, fever, and fatigue. Mean serum lactate, C-reactive protein (CRP), aspartate aminotransferase (AST), and alanine aminotransferase (ALT) levels were significantly higher in severe/critical patients compared to the other two groups (*P* < .05). severe acute respiratory syndrome coronavirus 2 negativity in control swabs (n=26) occurred at a mean of 10.6 ± 2.9 days after symptom onset. Forty-three patients (95.6%) were followed in the ward and 2 (4.4%) were admitted to the intensive care unit.

**Conclusion:** Children aged 0–18 years constituted a very small proportion of coronavirus disease 2019 reverse transcription-polymerase chain reaction -positive cases. Asymptomatic carriage of SARS-CoV-2 by a large proportion of children seems to be a major factor driving community spread. Some children with coronavirus disease 2019 may also present neurological findings. coronavirus disease 2019 infection is more severe in patients with comorbidities, and support therapy is important in these patients.

**Main points**

- The results of this study show that pediatric patients comprise a small proportion of all coronavirus disease 2019 (COVID-19) cases, family contact is an important source of infection.
- Coronavirus disease 2019 can also present with neurological findings in children.
- The fact that a large portion of patients were asymptomatic indicates that children are an important factor in community spread.
- Our findings indicate that chest tomography should be performed in symptomatic patients.
- The high prevalence of lactate dehydrogenase and lactate elevation in asymptomatic cases is noteworthy.
- Patients who had control swab tests showed severe acute respiratory syndrome coronavirus 2 reverse transcription-polymerase chain reaction negativity at a mean of 10.6 ± 2.93 days after symptom onset.
- Coronavirus disease 2019 infection is more severe in patients with comorbidities, and support therapy is important in these patients.

## Introduction

Coronavirus disease 2019 (COVID-19) is a respiratory disease that emerged in Wuhan, China in December 2019.^1^ It is caused by the novel severe acute respiratory syndrome coronavirus 2 (SARS-CoV-2). Following the rapid global spread of COVID-19, the World Health Organization (WHO) declared the situation a pandemic on March 11, 2020.^2^

Severe acute respiratory syndrome coronavirus 2 is transmitted mainly through contact and the respiratory system, but transmission via the digestive tract has also been reported.^3^ The most common symptoms of COVID-19 are fever, dry cough, myalgia, fatigue, and dyspnea.^4^ Cell entry of coronaviruses depends on the binding of the viral spike (S) proteins to cellular receptors and on S protein priming by host cell proteases. Severe acute respiratory syndrome coronavirus 2 uses the SARS-CoV angiotensin-converting enzyme 2 (ACE2) receptor for entry and the serine protease TMPRSS2 for S protein priming.^5^ angiotensin-converting enzyme 2 receptors are found at high densities in the lungs, liver, bile ducts, and proximal renal tubules. For this reason, multiorgan failure can occur in addition to respiratory signs and symptoms.^6^

According to WHO data, as of May 27, 2021, there had been 168 040 871 confirmed COVID 19 cases and 3 494 758 confirmed deaths worldwide.^7^ In Turkey, where the first case was also reported on March 11, 2020.^8^ There had been 5 220 549 confirmed COVID-19 cases and 46 970 deaths confirmed deaths as of May 27, 2021.^9^ According to these data, the mortality rate as of May 27, 2021, was 2.0% globally^7^ and 0.9% in Turkey.^9^

Although anecdotal evidence indicates that children are generally less affected by the disease, there are substantially fewer studies investigating the clinical course and treatment practices in children when compared with adult studies.^10-12^ The present study aimed to examine the clinical and laboratory characteristics of pediatric COVID-19 cases admitted to a single center.

## Materials and Methods

The study included 45 children who were hospitalized due to COVID-19 in the pediatrics ward of Atatürk University Faculty of Medicine between March and July 2020. COVID-19 was confirmed in all patients by positive SARS-CoV-2 reverse transcription-polymerase chain reaction (RT-PCR) test of naso-oropharyngeal swab samples. The samples were analyzed in the Erzurum provincial health laboratory, which is licensed for SARS-CoV-2 testing by the General Directorate of Public Health of the Turkish Ministry of Health.^13^ Patient files and medical records in the electronic database were analyzed retrospectively by two experienced physicians (G.M.A. and C.N.). The patients were grouped by age as <1 year, 1–5 years, 6–10 years, and 11–18 years. The study was approved by the Atatürk University Faculty of Medicine Ethics Committee (dated June 26, 2020, committee/decision number: 07/27).

### Definitions

The following definitions were used to evaluate the cases:

1. Coronavirus disease 2019-positive case: SARS-CoV-2 was detected by RT-PCR assay of an appropriately collected nasopharyngeal swab sample.^13^
2. Date of symptom onset: The first day that symptoms appeared.
3. Source case: Symptomatic/asymptomatic COVID-19-positive individual with whom the patient had contact, considering the incubation period.^13^
4. Time to SARS-CoV-2 RT-PCR negativity: Number of days from symptom onset to a negative result in follow-up swab tests, which were performed every 24–48 hours after day 7 of hospitalization.
5. Upper respiratory tract infection: Fever accompanied by clinical signs and symptoms including cough, nasal discharge, coryza, pharyngitis, tonsillitis, otitis media, and sinusitis.^14^
6. Discharge criteria: Negative SARS-CoV-2 test result for nasopharyngeal swab obtained from the upper respiratory tract, no fever for 72 hours, normal feeding, radiological and clinical improvement, and oxygen saturation 95% or higher in room air.^13,15^ In the early period of pandemics, for discharge, we tried to obtain a negative SARS-CoV-2 test result for nasopharyngeal swab, however, in the following period, this was abandoned.
7. In cases with pneumonia, clinical stage and chest computed tomography (CT) were evaluated according to the criteria in the COVID-19 guidelines issued by the Turkish Ministry of Health.^13^
8. Patients were classified as having mild, moderate, severe, and critical disease based on previous definitions of the clinical manifestations of COVID-19 in pediatric patients.^14,15^ Due to the small number of patients, mild and moderate cases were combined into the mild/moderate group, and severe and critical cases into the severe/critical group.

When patients met the discharge criteria, consent to comply with home isolation was obtained from their families and they were discharged. During the study, COVID-19 diagnosis, follow-up, and treatment were performed in accordance with the guidelines published by the COVID-19 Scientific Committee of the Turkish Ministry of Health.^13^

### Statistical Analysis

Continuous variables were expressed as mean ± standard deviation and categorical variables as number and percentage. Normality of data distributions was assessed with the Kolmogorov–Smirnov test. Analysis of variance followed by post hoc Tukey’s or Tamhane’s test was performed to compare means of continuous variables between more than two groups. Independent-samples *t* tests were used for comparisons of normally distributed continuous data between two groups. Nonparametric tests were used to compare continuous data that were not normally distributed. Categorical data were compared using chi-square or Fisher’s exact tests, as appropriate. All probabilities are two-tailed. *P* values <.05 were considered statistically significant. Analyses were performed using SPSS Statistics version 25.0 (IBM Corp., Armonk, NY, USA).

## Results

Of 853 patients admitted to the Atatürk University Faculty of Medicine between March and July 2020 with positive SARS-CoV-2 RT-PCR test of naso-oropharyngeal swab sample, 45 patients (5.2%) were aged between 0 and 18 years. The mean age of the pediatric group was 90.2 ± 67.5 months (range 7–24 months) and 23 (51%) were female.

**Figure 1.**
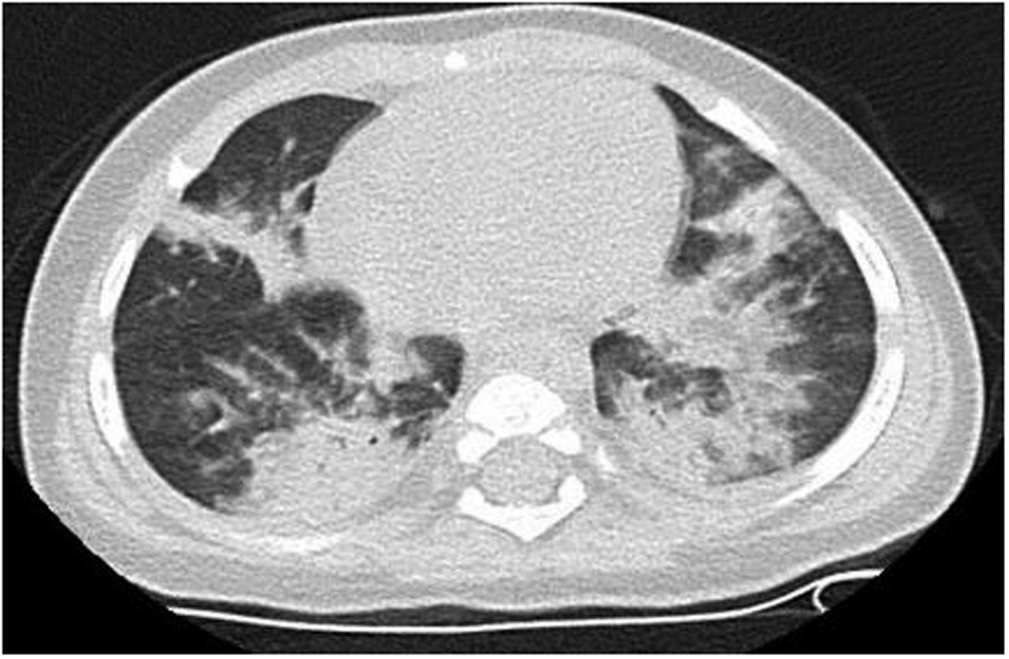
High-resolution computed tomography (HRCT) of an 11-month-old boy with epilepsy, hydrocephalus, cognitive/motor delay, sepsis, and COVID-19 pneumonia: Bilateral ground-glass opacities and areas of complete/incomplete consolidation containing sporadic air bronchograms in the basal segments and perihilar region that are more pronounced in the middle and lower zones of the lung parenchyma. COVID-19: coronavirus disease 2019.

**Figure 2. f2-eajm-54-2-173:**
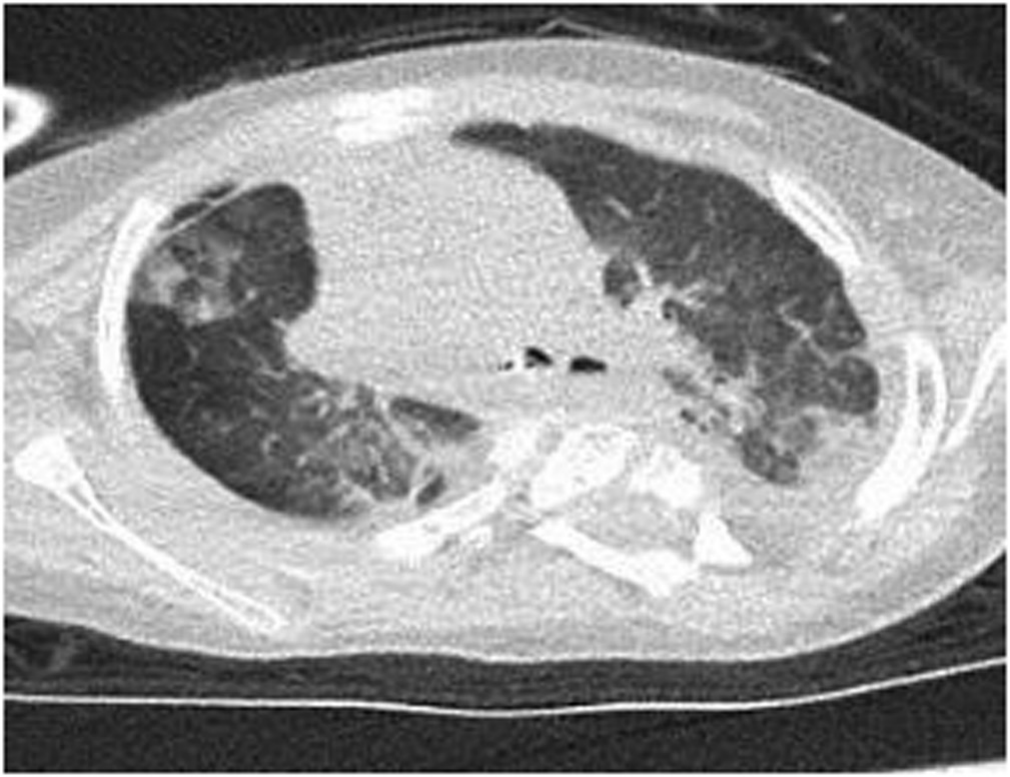
High-resolution computed tomography (HRCT) of a 3-year-old girl with VACTERL association, epilepsy, cognitive/motor delay, and COVID-19 pneumonia: Areas of complete/incomplete consolidation with sporadic air bronchograms in bilateral lung parenchyma and ground-glass opacities in the bilateral lung parenchyma. COVID-19: coronavirus disease 2019.

**Figure 3. f3-eajm-54-2-173:**
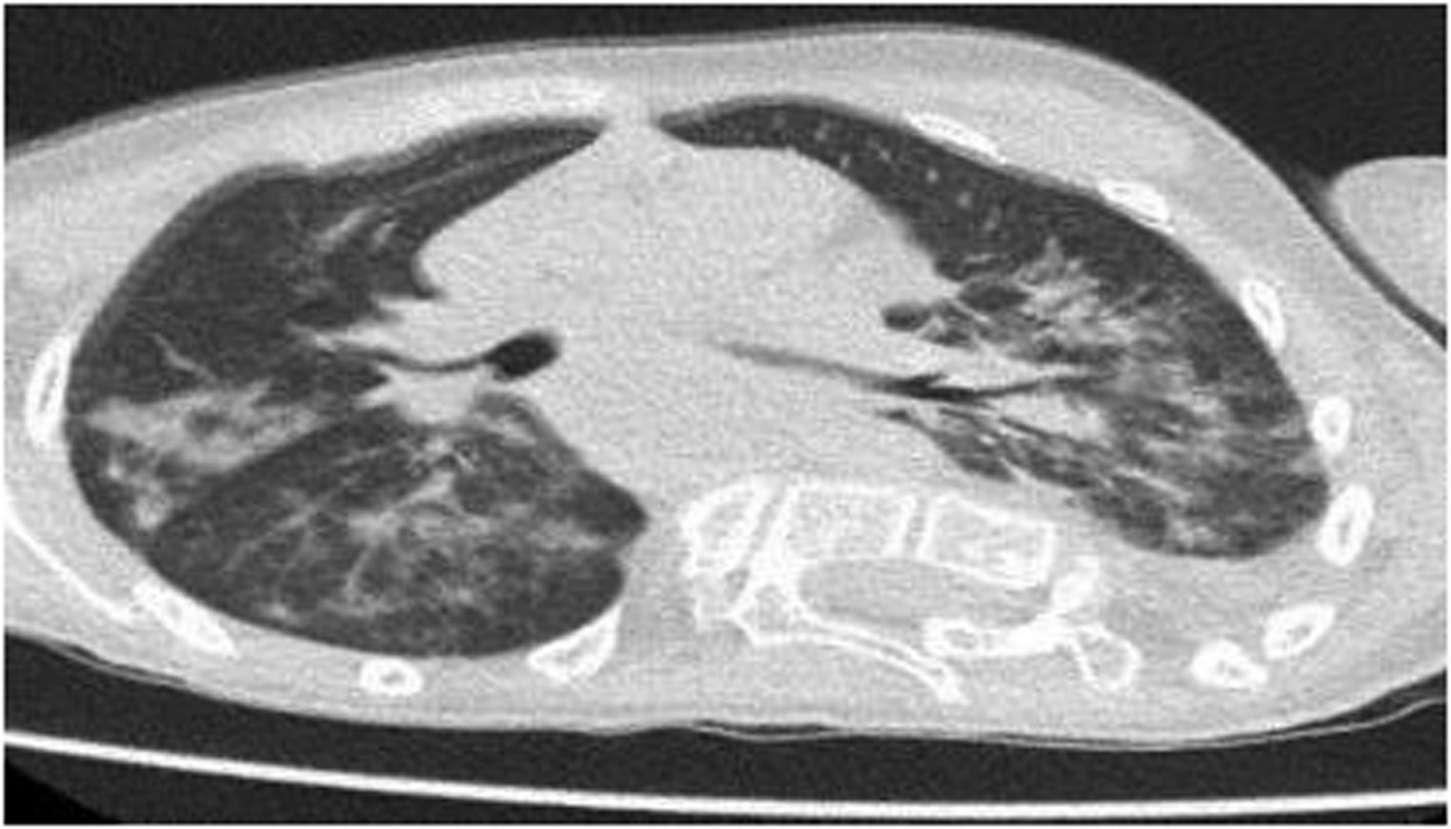
High-resolution computed tomography (HRCT) of a 12-year-old boy with Canavan disease, epilepsy, and COVID-19 pneumonia: Bilateral areas of diffuse alveolar consolidation and infiltration that are more pronounced in the upper lobes and sporadic ground-glass opacities. COVID-19: coronavirus disease 2019.

Clinical presentation was asymptomatic in 15 cases (33.3%), mild/moderate in 26 cases (57.8%), and severe/critical in 4 cases (8.9%). The patients’ demographic and epidemiological data according to COVID-19 clinical severity were presented in Table 1. The distribution of clinical severity differed significantly between age groups (*P* = .026). Most patients were in the 1–5 or 11–18 age groups. There were no neonates in the patient group. Three patients (6.6%) had chronic medical conditions that placed them in the high-risk group for COVID-19: epilepsy, hydrocephalus, and cognitive/motor delay (n=1) (Figure 1), VACTERL association, epilepsy, and cognitive/motor delay (n = 1) (Figure 2), and Canavan disease, cerebral palsy, and epilepsy (n = 1) (Figure 3). The majority of patients had contracted COVID-19 through household transmission.

Clinical features observed according to the clinical presentation were shown in Table 2. The mean time from symptom onset to hospital admission was 2.8 ± 1.8 (2.2–3.3) days. The prevalence of pneumonia increased significantly with the level of clinical severity (*P* < .001). The most common symptoms were cough, fever, and fatigue. Of the 30 symptomatic patients, signs and/or symptoms were consistent with lower respiratory tract infection in 15 (33.3%), upper respiratory tract infection in 12 (26.7%), and nonrespiratory infection in 3 patients (6.7%). Patients who had control swab tests (n=26) showed SARS-CoV-2 RT-PCR negativity at a mean of 10.6 ± 2.93 days after symptom onset. This time was longer in the severe/critical group, but not significantly (*P* > .05) (Table 2). PCR negativity time was 11.67 ± 3.42 days in girls and 9.79 ± 2.23 days in boys (*P* = .133). Length of hospital stay was significantly longer in the severe/critical group (*P* = .035). Length of stay was 9.96 ± 5.47 days for girls and 8.41 ± 5.11 days for boys (*P* = .406). Only 2 patients (4.4%) were admitted to the intensive care unit. The medications used during inpatient treatment are shown in Table 2.

Mean serum lactate, C-reactive protein (CRP), aspartate aminotransferase (AST), and alanine aminotransferase (ALT) levels were significantly higher in the clinically severe/critical group than in the asymptomatic and mild/moderate groups (*P* < .05). Mean serum troponin I, D-dimer, procalcitonin (PCT), and ferritin levels were markedly higher in the severe/critical group compared to the other groups, but there were no statistical differences (*P* > .05) (Table 3).

Of 37 patients who underwent chest x-ray, 6 (16.2%) had pathological findings suggestive of COVID-19. In contrast, 15 (40.5%) of 37 patients who underwent chest CT had pathological findings suggestive of COVID-19 (*P* = .006). The patients’ radiological data were summarized in Table 4. The distribution of chest CT findings according to clinical presentation is shown in Table 5. The frequency of specific CT findings increased significantly with the level of disease severity (*P* = .002). All patients were discharged in good condition and advised to complete a period of home isolation.

## Discussion

Coronavirus disease 2019 is milder in children than adults, although the mechanisms underlying this are not well understood.^16,17^ Studies from different countries have reported that children account for 1.7–4% of COVID-19 cases.^12,18,19^ A total of 853 patients were hospitalized in our center for COVID-19 between March and July 2020, of which 45 (5.2%) were pediatric patients. This study evaluated children with RT-PCR-confirmed SARS-CoV-2 infection who were hospitalized in a university hospital in the eastern region of Turkey. During the study period, all patients who were SARS-CoV-2 RT-PCR-positive were followed in hospital for isolation purposes, even if they were asymptomatic. Later, the Turkish Ministry of Health recommended hospitalizing only the patients who required respiratory support. For this reason, we believe the rate of 5.2% accurately represents the ratio of pediatric COVID-19 cases in our region. This rate was slightly higher than the literature data,^12,18,19^ which may be related to the large young population in our country.

In a study evaluating 1 49 082 pediatric cases in the United States, the mean age was 11 years and the largest age group was 15-17 years (32%).^12^ A recent study from the United States indicates the same result.^20^ In our study, the mean age was 90.2 ± 67.5 (7-204) months. The distribution of clinical severity differed significantly between the age groups (*P* = .026), but no age group had a predominant clinical presentation (Table 1).

Male sex was reported to be a risk factor among children admitted to intensive care.^10^ In contrast, other studies reported that male sex was not an independent risk factor for severe COVID-19.^20^ Effect of sex on clinical presentation is debatable, while some offer male sex as a risk factor for children admitted to intensive care.^11,21^ No mechanism has been proposed to explain the relationship between male sex and disease severity. The male-to-female ratio in the present study was 0.95:1, while 3 of the 4 patients with severe/critical diseases were male. However, sex distribution did not differ significantly according to the clinical presentation (χ^2^ = 5.109, *P* > .05). The small number of patients limits our ability to draw conclusions on this issue.

Literature indicates the role of asymptomatic children in transmission.^22^ Studies have reported that 13–27% of pediatric patients diagnosed with COVID-19 had the asymptomatic disease, while 8–10% had a severe disease or required intensive care support.^11,12,14,23^ One-third of the pediatric cases in our study, were asymptomatic. These cases were identified after a family member tested positive for COVID-19 or by screening. These cases were identified during family screening due to another index case. The high rate of asymptomatic infection in children seems to be one of the major factors driving community spread.

The most commonly reported sources of COVID-19 transmission are mother, father, or sibling (60%), while the rest (40%) are close nonfamily members or unknown.^11^ In the present study, the source of infection was family in 29 (64.4%) of the 45 cases. This demonstrates that close contact and cohabitation are important risk factors.

It has been suggested that COVID-19 is more severe among children with congenital or acquired comorbidities^3,11,24,25^ Oualha et al^26^ reported comorbidity in 70% of 27 clinically severe patients admitted to the intensive care unit. The most common reported comorbidities were immunosuppression (15.8%) and lung disease (12.5%).^27^ Childhood obesity is likely positively correlated with COVID-19 severity.^24,25,28^ As our study is retrospective, the heights of the patients was not available in some patients. So, a comparison in terms of body mass index could not be done. Of the 4 patients (8.8%) evaluated as clinically severe/critical in our study, 3 (75%) had comorbidities.

Pediatric COVID-19 studies have reported fever and cough as the most common symptoms.^11,12,23^ However, approximately 4-10% of infected children initially exhibit gastrointestinal symptoms such as diarrhea, abdominal pain, and vomiting.^23^ The most common symptoms in the present study were cough (51.1%) and fever (46.6%), while 4 patients (8.9%) presented with diarrhea. None of the patients had signs of renal or liver dysfunction.

Reports are accumulating of neurological involvement such as transverse myelitis in pediatric patients with COVID-19 infection.^29,30^ Two girls in our series, 5 and 13 years of age, complained of loss of smell and taste. Both patients showed no other clinical signs of COVID-19 during follow-up, and their smell and taste recovered after 2 weeks. Another 12-year-old girl presented with a loss of strength in her right arm and leg. The patient had suspected COVID-19 contact and was RT-PCR-positive for SARS-CoV-2. Her magnetic resonance imaging (MRI) findings were consistent with transverse myelitis in the cervical region. She responded to intravenous immunoglobulin (IVIG) and steroid therapy. These cases show that patients presenting with atypical neurological findings during the pandemic should be evaluated for COVID-19.

Pneumonia is an important component of COVID-19 infection. Although COVID-19 also involves extrapulmonary organs, COVID-19 is more severe in patients with serious lung involvement.^26^ Henry et al^31^ reviewed a large number of studies including a total of 66 pediatric patients ranging in age from 2 weeks to 17 years and reported that 72.7% of the cases were symptomatic and 53% showed radiological abnormalities. Pneumonia was detected in 15 (33.3%) of the patients in the present study.

It has been reported that chest x-ray has a limited ability to visualize lesions in children, and chest CT should be used in their diagnosis and follow-up.^3^ Early in the pandemic, CT imaging was performed in asymptomatic PCR-positive patients to evaluate for the presence of clinically silent pneumonia. However, this practice was later abandoned after updated guidelines stated it was not necessary. Our findings support these guidelines, as findings interpreted as consolidation were detected in only 1 of 12 asymptomatic patients on CT, and the frequency of pneumonia was shown to increase significantly with more severe clinical presentation (*P* < .001) (Table 5, Figures 1-4).

Some COVID-19 patients require intensive care. This rate is higher in adults and relatively low in children. In a comprehensive literature review evaluating 23 studies, risk factors for severe COVID-19 were age <1 year, presence of comorbid disease, older age, and lymphopenia. In the same study, it was reported that respiratory syncytial virus co-infection, immune system response, vaccination history, vitamin D levels, and genetic polymorphisms may be other factors associated with COVID-19 severity in children, but that data regarding these factors was insufficient.^21^ A multicenter COVID-19 study indicated that 5% of patients had concomitant infection with other viruses and that these patients showed a greater need for intensive care, respiratory support, and inotropic support.^11^ In the present study, level II intensive care was required by an 11-month-old patient with the comorbid disease and human metapneumovirus detected in a respiratory swab sample and by a 9-year-old patient with no comorbidity who was diagnosed with Kawasaki disease.

Different studies have reported negative SARS-CoV-2 RT-PCR results after 5 to 10 days of treatment.^10,23^ Among the patients in the present study who had control swab tests, the mean time to SARS-CoV-2 RT-PCR negativity was 10.6 ± 2.9 days after symptom onset. Mean test negativity time did not differ significantly according to disease severity (*P* > .05) but was observed to be longer in the severe/critical group (Table 2). This result indicates that more caution is warranted regarding the duration of transmissibility in severe cases.

The mean length of hospital stay was significantly longer for severe/critical patients than for asymptomatic patients (*P* = .037) but similar to that of the mild/moderate patients (*P* > .05). This finding suggests that the time to meet discharge criteria is similar among all symptomatic patients.

Although changes in laboratory parameters have been clearly described in adults diagnosed with COVID-19, experience is more limited in children. Some studies have reported normal or increased white blood cell (WBC) count, normal lymphocyte count, and elevated heart enzymes, liver enzymes, lactate dehydrogenase (LDH), CRP, PCT, D-dimer, and IL-10 levels in pediatric patients with severe/critical COVID-19.^23,27,31^ However, it was also reported that a model of laboratory values based on clinical severity in pediatric COVID-19 cannot be created based on the available literature data.^21^ The severe/critical patient group in the present study was found to have elevated CRP, D-dimer, troponin I, lactate, LDH, AST, ALT, PCT, and prothrombin time and decreased WBC, calcium, and albumin levels. Patients with high troponin I values had normal echocardiographic findings.

Statistical analysis showed that severe/critical cases had significantly higher mean serum lactate, CRP, AST, and ALT levels and significantly lower mean serum total calcium and albumin levels compared to asymptomatic and mild/moderate cases (*P* < .05). Our results showed that asymptomatic patients frequently showed elevation of LDH (14/15) and lactate (7/14). These results suggest that serum LDH and lactate levels can be used as preliminary markers to determine the need for isolation and swab PCR test in pediatric patients who present to emergency clinics without symptoms consistent with COVID-19 infection (Table 3).

Henry et al^31^ in their meta-analysis suggested serial monitoring of PCT for secondary infections in COVID 19 disease. Other authors have stressed the importance of CRP and PCT elevation as indicators of concurrent bacterial infection in pediatric patients.^3,21^ In the present study, a sudden increase in CRP and PCT levels and growth of *Acinetobacter* in blood culture were observed in one patient with COVID-19 pneumonia. This supports the use of CRP and PCT to detect newly emerging bacterial infections in pediatric COVID-19 patients. A sudden increase in CRP and PCT levels and growth of *Acinetobacter* in blood culture after central catheter insertion was observed in one patient with COVID-19 pneumonia. This may suggest that CRP and PCT may be used in the detection of newly emerging bacterial infections in pediatric COVID-19 patients.

The use of antibiotics for bacterial superinfections in pediatric patients with COVID-19 has been referred to in many studies.^17^ When viral infections such as influenza or COVID-19 impair host defenses, microorganisms such as *Streptococcus pneumoniae* and* Staphylococcus aureus* can cause fatal secondary bacterial pneumonia in patients with comorbidities or healthy individuals.^25^ Some patients die from bacterial co-infection rather than the virus itself. Therefore, bacterial co-infection and secondary bacterial infection are considered critical risk factors in COVID-19 severity and mortality.^26^

Hydroxychloroquine, lopinavir/ritonavir, favipiravir, remdesivir, tocilizumab, anakinra, and ribavirin have been used in the treatment of COVID-19.^11,32^ Other recommended treatment approaches include short-term low-to-moderate dose corticosteroids, IVIG, interferons, and COVID-19 convalescent plasma.^33,34^ The combined use of hydroxychloroquine and azithromycin was suggested to reduce the viral load of SARS-CoV-2.^17^ Hydroxychloroquine was removed from the treatment recommendations in the last guideline of the Ministry of Health of the Republic of Turkey.^28^ With respect to clinical management, none of the therapies instituted in the treatment of children with severe COVID-19 disease have been demonstrated to improve outcomes in randomized trials; therefore, a recommendation regarding their use is challenging.^27^ At present, there is no antiviral treatment for SARS-CoV-2 with proven efficacy.^27,28^ For pediatric patients, antiviral treatment is more preferred for severe cases.^13,28^ In our study, antiviral treatment was initiated in the severe/critical patients in our series (lopinavir/ritonavir + hydroxychloroquine in 2, hydroxychloroquine in 2). In all patients, empirical treatment with oseltamivir was started and was discontinued when influenza was not detected in the respiratory panel.

In a study from Turkey, it was emphasized that COVID-19 is mild in children and treatment is primarily supportive care.^35^ The results of a multicenter study showed that 87% of patients did not require respiratory support at any stage, while 5% of the patients required continuous positive airway pressure and 4% required mechanical ventilation.^11^ Similarly, 41 (91.1%) of our patients did not require respiratory support at any stage, and 4 patients (8.8%) required oxygen support. Mechanical ventilation was not needed in any case. Our findings support existing data indicating that COVID-19 is relatively milder in children.

## Study Limitations

The small number of patients included in this study precludes definitive conclusions on some topics. The very small number of severe/critical patients, in particular, may have influenced the results. Nevertheless, this study provides important information about the course of COVID-19 in pediatric patients.

In conclusion, the results of this study show that pediatric patients comprise a small proportion of all COVID-19 cases. COVID-19 infection is more severe in patients with comorbidities, and supportive therapy is important in these patients. As vaccine and specific drug studies continue, more extensive multicenter studies are needed to clarify approaches to the diagnosis, follow-up, and treatment of pediatric COVID-19 patients.

## Figures and Tables

**Table 1.** Demographic and Epidemiological Data of Pediatric COVID-19 Patients According to Clinical Presentation

**Variables**		**Disease Severity**			**Total n=45**	*P*
**Asymptomatic n=15** **(33.3%)**	**Mild/ Moderate n=26** **(57.8%)**	**Severe**/ **Critical n=4 (8.9%)**
**Demographic, epidemiological data**						
	**Age (n, %)**					
	<1 year	4 (57.0)	2 (28.6)	1 (14.4)	7 (15.5)	.026
	**1-5 years**	5 (33.3)	9 (60.0)	1 (6.70)	15 (33.3)	
	**6-10 years**	4 (80)	0	1 (20)	5 (11.2)	
	**11-18 years**	2 (11.1)	15 (83.4%)	1 (5.5)	18 (40.0)
Mean±SD (months)	57.4 ± 43.7	111.5 ± 73.0	75.5 ± 62.5	90.2 ± 67.5	
	**Sex (n, %)**					
	**Female**	5 (21.7)	17 (74.0)	1 (4.3)	23 (51.1)	.078
**Male**	10 (45.4)	9 (41.0)	3 (13.6)	22 (48.9)
**Comorbidity**		0	0	3 (75)	4 (8.9)	-
	**Contact source**					
	**Immediate family**	12 (41.3)	16 (55.2)	1 (3.5)	29 (64.4)	.079
	**Other relative**	0	4 (100)	0	4 (8.9)	
**Unknown**	3 (25)	6 (50)	3 (25)	12 (26.7)

COVID-19: coronavirus disease 2019.

**Table 2.** Clinical Data of Patients According to Disease Severity

		**Disease Severity**			**Total n (%)**	*P*
**Asymptomatic n=15 (33.3%)**	**Mild/Moderate n=26 (57.8%)**	**Severe/Critical n=4 (8.9%)**
**Clinical Data**						
	**Pneumonia**					
	None	14 (46.6)	16 (53.4)	0	30 (66.6)	<.001
	Mild	1 (1.3)	7 (8.7)	0	8 (17.9)	
	Severe	0	3 (60)	2 (40)	5 (11.1)	
Very severe	0	0	2 (100)	2 (4.4)
	**Symptoms**					
	Cough	-	19 (82.6)	4 (17.4)	23 (51.1)	
	Fever >37.5°C	-	17 (81.0)	4 (19.0)	21 (46.7)	
	Malaise	-	8 (66.6)	4 (33.4)	12 (26.7)	
	Headache	-	4 (80)	1 (20)	5 (11.1)	
	Nausea/vomiting	-	4 (80)	1 (20)	5 (11.1)	
	Dyspnea	-	0	4 (100)	4 (8.9)	
	Diarrhea	-	3 (75)	1 (25)	4 (8.9)	
	Muscle pain	-	3 (75)	1 (25)	4 (8.9)	
	Conjunctivitis	-	2 (66.6)	1 (33.4)	3 (6.7)	
	Loss of smell/ taste	-	2 (100)	0	2 (4.4)	
	Hemiplegia	-	1 (100)	0	1 (2.2)	
	Chest pain	-	1 (100)	0	1 (2.2)	
Maculopapular rash	-	0	1 (100)	1 (2.2)
	**Treatment**					
	Other antibiotics	0	9 (69)	4 (31)	13 (28.9)	
	Azithromycin	0	11 (73.3)	4 (26.7)	15 (33.3)	
	Oseltamivir	0	6 (75)	2 (25)	8 (18)	
	Hydroxychloroquine	0	0	4 (100)	4 (8.9)	
	Ritonavir + lopinavir	0	0	2 (100)	2 (4.4)	
	Intravenöz Immunoglobulin	0	1 (50)	1 (50)	2 (4.4)	
	Oxygen via mask	0	0	1 (100)	1 (2.2)	
High-flow oxygen	0	0	3 (100)	3 (6.6)
**Ward patients, n (%)**		15 (100)	26 (100)	2 (50)	43 (95.6)	-
**Intensive care patients, n (%)**		0	0	2 (50)	2 (4.4)	-
		Mean±SD **(n)**	Mean±SD **(n)**	Mean±SD **(n)**		
**Mean ward stay (days)**		8±4.95(15)	8.96±5.17(26)	15.2±4.11(4)	9.2±5.29	*
**Time to SARS-CoV-2 PCR-negativity**		9.69±2.13(13)	11.4±3.5(11)	12.5 (2)	10.6±2.93(26)	>.05

*Mean length of stay was significantly longer the severe/critical patient group than the asymptomatic patient group (*P*=.037).

PCR: polymerase chain reaction.

**Table 3.** Comparison of the Patients’ Laboratory Data According to Clinical Severity

**Laboratory Parameter**	**Disease Severity**			**Total**	*P*
**Asymptomatic** **n=15** **(33.3%)**	**Mild/ Moderate** **n=26** **(57.8%)**	**Severe/ Critical** **n=4** **(8.9%)**
LDH: N tested/n > 248 U/L	14/14	24/16	4/4	42/34	>.05
Lactate: N tested/n > 1.6 mmol/L	14/7	21/9	4/4	39/20	*
CRP: N tested/n > 3.14 mg/L	15/1	23/9	4/4	42/14	*
Aspartate aminotransferase: N tested/n > 45 U/L	15/3	26/4	4/4	45/11	*
D-dimer: N tested/n > 500 ng/ml	15/1	26/6	3/3	43/10	>.05
Calcium: N tested/n < 8.8 mg/dl	15/2	25/2	4/3	44/7	*
Prothrombin time: N tested n > 16 s	11/1	19/3	4/3	34/7	>.05
Lymphocytes: N tested/n <1.1 × 10**3**/μL	15/1	26/4	4/1	45/6	>.05
WBC: N tested/n < 4 × 10**3**/μL	15/0	26/2	4/2	45/4	>.05
Platelets: N tested/n < 150 × 10**3**/μL	15/1	26/0	4/3	45/4	>.05
Troponin I: N tested/n > 11.6 ng/L	14/0	20/1	3/3	37/4	>.05
Alanine aminotransferase: N tested/n > 45 U/L	15/0	26/1	4/3	45/4	*
Albumin: N tested/n < 3.5 g/dL	15/0	25/0	4/4	44/4	*
Procalcitonin: N tested/n > 2 ng/mL	10/0	18/0	3/2	31/2	>.05
Ferritin: N tested/n > 306 ng/mL	13/0	16/0	3/2	32/2	>.05

*The clinically severe/critical group differed significantly from the other two groups (*P* ≤ .05). There was no significant difference between the asymptomatic and mild/moderate groups (*P* > .05).

**LDH: lactate dehydrogenase, CRP, C-reactive protein, WBC, white blood cell.**

**Table 4.** Radiological Findings

N=45		n (%)
**Underwent chest x-ray**		37 (82.2)
**Pathology on chest x-ray**		6 (16.2)
**Underwent chest CT**		37 (82.2)
**Chest CT consistent with/suggestive of COVID-19**		15 (40.5)
	Pulmonary ground-glass opacities	10 (27.1)
	Consolidation	5 (13.5)
Normal	22 (59.4)

**COVID-19:** coronavirus disease 2019.

**Table 5.** Distribution of Chest Tomography Findings According to Clinical Severity

		**CT Findings**			*P*
**Normal**	**Consolidation**	**Ground-Glass Opacities**
**Clinical severity**					
	Asymptomatic (n = 12)	11	1	0	.002
	Mild/moderate (n = 21)	11	4	6	
Severe/critical (n = 4)	0	0	4
**Total**		22	5	10	

**Figure 4.**
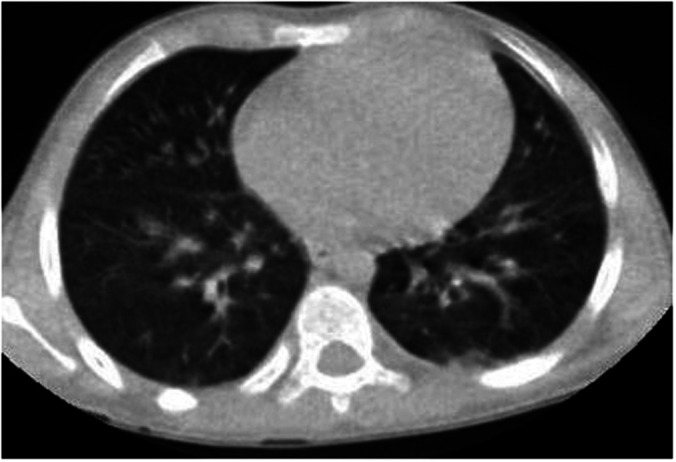
High-resolution computed tomography (HRCT) of a 9-year-old male patient with Kawasaki disease and COVID-19 pneumonia: Passive atelectatic changes on the right, ground-glass opacities near the pleural surface bilaterally and patchy ground-glass opacities in the middle lobe of the right lung. COVID-19: coronavirus disease 2019.
